# Research Advances in Drug Resistance Mechanisms to Anti-HER2 Therapy in HER2-Positive Breast Cancer

**DOI:** 10.32604/or.2026.085387

**Published:** 2026-07-16

**Authors:** Chunwei Huang, Jingyi Kong, Hangxing Ren, Wanchen Zhang, Shi Jiang, Xianneng Sheng

**Affiliations:** Department of Breast Surgery, The First Affiliated Hospital of Ningbo University, Ningbo, China

**Keywords:** HER2-positive, breast cancer, anti-HER2 therapy, resistance mechanisms

## Abstract

HER2-positive breast cancer accounts for 15–20% of all breast cancer cases. Although the development of monoclonal antibodies (e.g., trastuzumab, pertuzumab), tyrosine kinase inhibitors (e.g., lapatinib, pyrotinib), and antibody-drug conjugates (e.g., T-DM1, trastuzumab deruxtecan) has greatly improved patient prognosis, primary or acquired resistance to anti-HER2 therapy remains a major clinical challenge, leading to treatment failure and disease progression. Recent research has elucidated diverse resistance mechanisms, including HER2 signaling pathway aberrations (such as receptor mutations, alternative splicing, and bypass activation), tumor microenvironment remodeling (involving immunosuppressive cells, metabolic reprogramming, and immune checkpoint molecules), and ADC-specific resistance (impaired internalization, lysosomal dysfunction, payload efflux, and ferroptosis blockade). However, existing reviews primarily focus on trastuzumab and classical signaling pathways, with insufficient integration of ADC-specific mechanisms or microenvironmental immune evasion. Furthermore, the translation of mechanistic discoveries into clinical strategies remains weak, and a systematic summary of validated biomarkers (e.g., PIK3CA mutations, PTEN loss, p95HER2, ADAR1, HLA-G) and related clinical trials is lacking. The purpose of this review is threefold: (1) to systematically integrate recent advances in anti-HER2 resistance mechanisms from three perspectives—HER2 signaling abnormalities, tumor microenvironment remodeling, and ADC-specific barriers; (2) to provide an evidence-based framework for target prioritization by categorizing mechanisms according to their validation stage (clinically validated, substantial *in vivo* evidence, or *in vitro* studies only); and (3) to summarize current biomarker-driven clinical trials and emerging therapeutic strategies, including combination immunotherapy, CDK4/6 inhibitors, PI3K PROTACs, and cold atmospheric plasma. Ultimately, this review aims to bridge the gap between basic research and clinical practice, offering practical guidance for overcoming anti-HER2 resistance through precision combination strategies in HER2-positive breast cancer.

## 1 Introduction

### 1.1 Epidemiology and Clinical Characteristics of HER2-Positive Breast Cancer

According to the latest data from the International Agency for Research on Cancer in 2022, breast cancer (BC) has become the second most common cancer globally after lung cancer and the most prevalent malignant tumour among women [1]. Approximately 15–20% of breast cancer cases exhibit amplification of the human epidermal growth factor receptor 2 (HER2) gene or overexpression of its protein, classifying them as HER2-positive. These tumours are characterized by high aggressiveness and poor prognosis [2,3].

### 1.2 The HER2 Signalling Pathway and Its Key Downstream Pathways

The ERBB (Erythroblastic Leukaemia Viral Oncogene Homolog) family constitutes a crucial family of receptor tyrosine kinases (RTKs) that play a central role in cellular signal transduction, regulating biological processes including cell proliferation, differentiation, migration, and survival [4]. This family includes four members: EGFR (HER1), HER2, HER3, and HER4. All share a similar structure, featuring an extracellular ligand-binding domain, a transmembrane domain, and an intracellular tyrosine kinase domain. Notably, HER2 overexpression or gene amplification constitutes a key driver of breast cancer and forms the molecular basis for anti-HER2 therapy. HER2 overexpression activates signalling pathways such as PI3K/AKT/mTOR through homodimerisation or heterodimerisation with EGFR, HER3, and others, thereby promoting tumour cell proliferation, enhanced metabolism, and invasiveness [5].

#### 1.2.1 The PI3K/AKT/mTOR Signalling Pathway

The PI3K/AKT/mTOR pathway transmits growth signals through a cascade of phosphorylation events, regulating the survival, metabolism, and metastasis of breast cancer cells [6]. When ligands such as insulin or insulin-like growth factor bind to cell membrane receptors, they activate phosphoinositide 3-kinase (PI3K). PI3K then catalyzes the conversion of phosphatidylinositol bisphosphate (PIP_2_) to phosphatidylinositol trisphosphate (PIP_3_). PIP_3_ binds to the PH domain of protein kinase B (AKT), recruiting it to the membrane where mTOR complex 2 (mTORC2) phosphorylates AKT at Ser473. Simultaneously, PIP_3_ anchors phosphoinositide-dependent protein kinase 1 (PDK1) to the membrane, enabling PDK1 to phosphorylate AKT at Thr308. Activated AKT subsequently regulates the cell cycle, growth, proliferation, and energy metabolism [5]. Consequently, the persistent activation of the PI3K/AKT/mTOR signalling pathway resulting from HER2 overexpression constitutes a significant driver of breast cancer progression.

#### 1.2.2 MAPK/ERK Signalling Pathway

The MAPK/ERK signalling pathway is one of the core regulatory pathways governing cell proliferation, differentiation, survival, and migration [7]. In HER2-positive breast cancer, this pathway is abnormally activated, driving tumour progression. Upon phosphorylation and activation, the HER2 receptor recruits guanine nucleotide-exchange factors (SOS) via adaptor proteins (e.g., GRB2, SHC). SOS then activates RAS proteins (e.g., KRAS, NRAS) by facilitating the exchange of GDP for GTP. Activated RAS binds and activates RAF kinases (e.g., BRAF, CRAF), which phosphorylate and activate MEK. MEK, in turn, phosphorylates ERK, fully activating it and initiating the RAF-MEK-ERK cascade.

### 1.3 Developments in Anti-HER2 Targeted Therapy

The rationale for anti-HER2 therapy in breast cancer primarily rests upon targeting the biological characteristics of HER2 and its role in tumour initiation and progression, employing multiple mechanisms to block or disrupt the HER2 signalling pathway. With the iterative development of anti-HER2 targeted therapies—from the introduction of the first HER2 monoclonal antibody, trastuzumab [8], to the clinical application of pertuzumab, the small-molecule tyrosine kinase inhibitor lapatinib, and antibody-drug conjugates such as trastuzumab emtansine (T-DM1)—anti-HER2 treatment has significantly improved overall survival rates for patients with HER2-positive breast cancer [9] (Table 1). Trastuzumab binds to the extracellular domain of HER2, thereby blocking its dimerisation with other HER family members and inhibiting signal transduction [10]. Additionally, the antibody’s Fc fragment binds to Fcγ receptors on immune effector cells (such as natural killer cells), activating antibody-dependent cellular cytotoxicity (ADCC) effects to induce the killing of antibody-labelled tumour cells by the immune system [11]. APT was an open-label, single-arm Phase II clinical trial that enrolled a total of 410 patients with HER2-positive breast cancer who were lymph node-negative and had tumours measuring ≤3 cm. The study showed that treatment with paclitaxel in combination with trastuzumab for 12 weeks resulted in a 10-year disease-free survival (iDFS) rate of 91.3% and a 10-year overall survival rate of 94.3% [12]. Tyrosine kinase inhibitors competitively bind to the ATP-binding site within the intracellular domain of the HER2 receptor, thereby inhibiting its tyrosine kinase autophosphorylation and activation [13]. This ultimately blocks key downstream signalling pathways, achieving the suppression of proliferation, invasion, and metastasis in HER2-positive breast cancer cells. Such small-molecule drugs (e.g., pyrotinib, lapatinib, neratinib, etc.) can simultaneously inhibit other members of the HER family and penetrate the cell membrane to act on intracellular domains. This characteristic renders them potentially effective in certain patients who have developed resistance to large-molecule monoclonal antibodies (such as trastuzumab) due to structural alterations in the HER2 receptor (e.g., the truncated form p95HER2) or abnormal activation of downstream signalling pathways [14]. In the PHILA study, the combination of pyrotinib with trastuzumab and docetaxel achieved a median progression-free survival (PFS) of 24.3 months, offering a new treatment option for first-line therapy in advanced disease [15]. Antibody-drug conjugates (T-DM1) represent a method for the targeted delivery of cytotoxic drugs to cancer cells. Their mechanism involves the antibody portion specifically binding to HER2-positive cancer cells, facilitating the endocytosis of the cytotoxic drug into the tumour cell. T-DM1 delivers the microtubule inhibitor DM1, which inhibits microtubule polymerisation and induces cancer cell apoptosis. Concurrently, DM1’s action does not compromise the anti-tumour efficacy of the antibody (trastuzumab) [16]. The KATHERINE study demonstrated that T-DM1 reduces the risk of invasive disease recurrence by 50% compared with trastuzumab (3-year iDFS rates of 88.3% vs. 77.0%), establishing it as the standard of care for this patient population [17].

**Table 1:** Comparison of commonly used anti-HER2 drugs.

Drug Name	Drug Category	Mechanism of Action	Main Mechanisms of Resistance	Clinical Limitations
Trastuzumab	monoclonal antibody	By binding to the extracellular domain IV of HER2, it blocks HER2 homodimerisation whilst mediating immune-mediated cell killing via ADCC	1. Masking or truncation of the HER2 extracellular domain (e.g., p95HER2); 2. Bypass activation of the HER family (HER3/NRG1); 3. Activation of downstream pathways (PIK3CA mutations, PTEN loss); 4. Immunosuppressive microenvironment (TAM, HLA-G)	Cardiotoxicity; high incidence of primary and acquired resistance (ORR of approximately 35% when used as monotherapy)
Pertuzumab	monoclonal antibody	By binding to the HER2 extracellular domain II, it blocks HER2/HER3 heterodimerisation	1. Absence or downregulation of HER2 expression; 2. Activation of bypass pathways (c-MET, EGFR); 3. Sustained activation of the downstream PI3K/AKT pathway	Usually used in combination with trastuzumab; has limited efficacy when used alone; diarrhoea, neutropenia
Lapatinib	Small-molecule TKI (reversible, EGFR/HER2 dual-targeted)	By competitively binding to the intracellular ATP-binding sites of HER2/EGFR, it inhibits tyrosine kinase autophosphorylation and downstream signalling	1. HER2 truncation mutations (p95HER2 retains kinase activity); 2. HER3/NRG1 feedback activation; 3. Activation of compensatory signalling pathways (SRC/STAT3, YAP1); 4. Upregulation of drug efflux pumps (ABCG2)	Diarrhoea, rash; limited ability to cross the blood-brain barrier; less effective than newer-generation TKIs
Neratinib	Small-molecule TKIs (irreversible, pan-HER)	Binds irreversibly to the intracellular kinase domains of HER1/HER2/HER4, permanently inhibiting phosphorylation	1. HER2 point mutations (e.g., V777L); 2. Bypass activation (MET, IGF-1R); 3. HER3 reactivation following resistance	Severe diarrhoea, liver damage
Trastuzumab	Small-molecule TKIs (irreversible, pan-HER)	Irreversibly inhibits HER1/HER2/HER4 whilst simultaneously inhibiting the downstream AKT/ERK pathways	1. Mutations in the HER2 binding site; 2. Compensatory PI3K/AKT activation (PIK3CA mutations); 3. Bypass activation	Diarrhoea and hand-foot syndrome
T-DM1 (Trastuzumab Emtansine)	ADC (microtubule inhibitor DM1)	Trastuzumab targets HER2; following endocytosis, it releases DM1, which inhibits microtubule polymerisation and induces apoptosis	1. Downregulation of HER2 expression/heterogeneity; 2. Impaired endocytic/lysosomal transport (lysosomal dysfunction); 3. Upregulation of the drug efflux pump (MDR1); 4. Microtubule pathway mutations	Thrombocytopenia, hepatotoxicity; weak bystander effect; ineffective against tumours with low HER2 expression
T-DXd (Trastuzumab Deruxtecan, DS-8201)	ADC (topoisomerase I inhibitor DXD)	Trastuzumab targets HER2; upon endocytosis, it releases DXD, which inhibits topoisomerase I and induces DNA damage; it exhibits a strong bystander effect	1. Activation of the iron-dependent death inhibition pathway (crVDAC3/HSPB1); 2. Abnormal lysosomal function (altered cathepsin activity); 3. TOP1 mutations; 4. Upregulation of drug efflux pumps (ABCG2, ABCB1); 5. Very low HER2 expression	Interstitial pneumonia; nausea, bone marrow suppression; relatively high cost

Building upon T-DM1, the development of DS-8201 (T-DXd) represents a novel advancement in anti-HER2 therapy. It comprises a humanised anti-HER2 antibody, an enzyme-cleavable linker, and a novel topoisomerase I inhibitor. The antibody portion of DS-8201 specifically binds to HER2 receptors on tumour cell surfaces, promoting internalisation. The linker is cleaved in lysosomes, releasing DXD. DXD induces DNA damage by inhibiting topoisomerase I, leading to cell cycle arrest and apoptosis. Furthermore, DXD exhibits membrane permeability, diffusing to adjacent tumour cells (the “bystander effect”), thereby demonstrating significant antitumour activity even against tumours with low or heterogeneous HER2 expression. This mechanism enables DS-8201 to deliver superior efficacy in both HER2-positive, T-DM1-insensitive breast cancer and HER2-low breast cancer [18,19,20]. In the long-term survival analysis of the DESTINY-Breast03 trial (median follow-up 41 months), DS-8201 demonstrated significantly superior efficacy and durable survival benefit compared to T-DM1. The median progression-free survival (PFS) in the DS-8201 group was 29.0 months, substantially exceeding the 7.2 months observed in the T-DM1 group (HR = 0.30) [21]. In the DESTINY-Breast04 trial, DS-8201 significantly extended progression-free survival (10.1 vs. 5.4 months) and overall survival (23.9 vs. 17.5 months) compared to chemotherapy in patients with HER2-low advanced breast cancer, reducing the risk of disease progression or death by approximately 50% and the risk of death by 36%. This efficacy was observed regardless of hormone receptor status [22]. In the DESTINY-Breast09 trial evaluating first-line treatment for HER2-positive advanced breast cancer, DS-8201 combined with pertuzumab significantly extended progression-free survival (PFS) compared to standard triple therapy (chemotherapy plus dual-targeted therapy) (40.7 vs. 26.9 months), reducing the risk of disease progression by 44% and achieving a higher objective response rate (85.1% vs. 78.6%) [23]. This establishes it as a potential new standard first-line treatment regimen.

### 1.4 The Challenge of Resistance to Anti-HER2 Therapy

Despite these advances, primary or acquired resistance remains a major clinical challenge. Studies indicate that approximately 30–50% of advanced patients exhibit primary resistance to trastuzumab monotherapy [24], Furthermore, among HER2-positive metastatic breast cancer patients treated with T-DM1, median progression-free survival (PFS) was only 9.6 months (EMILIA trial) [25], suggesting widespread acquired resistance. Following disease progression due to resistance, patients face limited treatment options and significantly reduced survival.

### 1.5 Research Objectives

In recent years, reviews have systematically summarised the mechanisms of resistance to HER2-targeted therapy; however, most have focused on trastuzumab and classical signalling pathways, with insufficient exploration of the specific mechanisms of resistance to ADC drugs such as T-DM1 and T-DXd (e.g., internalisation barriers and lysosomal dysfunction). Furthermore, resistance mediated by the tumour microenvironment is often described in a fragmented manner in the existing literature, lacking effective integration with clinical strategies. Additionally, systematic analysis of the transition from mechanisms to clinical translation remains weak, with a lack of summaries regarding validated biomarkers such as PIK3CA mutations, PTEN loss, and p95HER2, as well as related clinical trials.

To address these gaps, the present review has three primary objectives: (1) to systematically integrate the current understanding of resistance mechanisms arising from HER2 signalling pathway abnormalities, tumour microenvironment remodelling, and ADC-specific resistance (e.g., impaired internalisation, lysosomal dysfunction, and payload efflux); (2) to provide an evidence-based framework for target prioritisation by grading each mechanism according to its validation level (clinically validated, substantial *in vivo* evidence, or *in vitro* studies only); and (3) to focus on clinical translation and precision treatment strategies, offering practical guidance for overcoming resistance to anti-HER2 therapy in HER2-positive breast cancer.

## 2 Mechanisms of Drug Resistance Mediated by Abnormal HER2 Signalling Pathways

### 2.1 HER2 Expression Alterations Mediating Drug Resistance

#### 2.1.1 Low HER2 Expression and the Absence or Mutation of the HER2 Receptor

At the genetic level, Simonova et al. employed methylation-sensitive restriction enzyme PCR to analyse 5′-Cytosine-Phosphate-Guanine-3′ (CpG) methylation within the promoter regions of multiple genes, including MMP2 and MMP23B. Results indicated that CpG demethylation in the promoter regions of MMP2, MMP23B, MMP24, MMP25, and MMP28 was associated with HER2 low expression in tumours. Furthermore, at least two sample groups exhibiting abnormally hypermethylated MMP genes showed significant enrichment of HER2-positive tumours. These findings collectively suggest that HER2 low expression may correlate with promoter region methylation [26].

Clinical trials have shown that the addition of trastuzumab to chemotherapy does not improve invasive disease-free survival (IDFS), distant recurrence-free survival (DRFI) or overall survival (OS) in patients with HER2-low breast cancer [27]. In a study by Hirotsu et al., 20 patients with HER2-positive breast cancer who relapsed after trastuzumab treatment were identified. Among these, six patients had metastatic sites, excluding brain metastases, resistant to trastuzumab. Among these six patients, one exhibited the HER2 V777L mutation both before and after trastuzumab treatment, whereas this mutation was absent in the fourteen patients who responded to trastuzumab. This suggests that the HER2 V777L mutation may underlie trastuzumab resistance and could serve as a predictive biomarker for such resistance [28].

#### 2.1.2 Upregulated HER2 Expression

At the transcriptional level, Liu et al. discovered that the extracellular lncRNA LINC00969 can upregulate HER2 protein expression at the post-transcriptional level, suggesting its potential role in inducing drug resistance. They demonstrated that LINC00969 binds to HuR (human antigen R) to stabilize HER2 mRNA, thereby promoting trastuzumab resistance in HER2-positive breast cancer cells [29]. 

At the translational level, research by Liu et al. indicates that the histone demethylase PHF8 synergises with the HER2 signalling pathway via epigenetic mechanisms in HER2-positive breast cancer: on the one hand, PHF8 promotes HER2 transcriptional expression by binding to the HER2 promoter region; on the other, HER2 signalling upregulates PHF8 expression, forming a positive feedback loop [30].

#### 2.1.3 Splicing Variants of the HER2 Gene

##### The Emergence of p95HER2 Splice Variants and Their Clinical Significance

Splicing variants of the HER2 gene result in the deletion of the transmembrane domain, yielding p95HER2, which contains only the intracellular kinase domain. Such variants evade targeted therapy by lacking the trastuzumab binding epitope, yet retain kinase activity, thereby sustaining downstream signalling activation [31]. 

A retrospective study of clinical patient data has shown that tumours expressing p95HER2 often develop resistance to trastuzumab [32]. 

##### Epigenetic Regulation of the HER2 Signalling Pathway by the MEL-18/ADAM10 Axis

At the genetic level, Lee et al. discovered that the polycomb group gene MEL-18 synergises with polycomb proteins (PcG, a crucial class of epigenetic regulators) to suppress ADAM10 transcriptional activity within its promoter region, thereby reducing the release of HER ligands such as HB-EGF and NRG1. In the absence of MEL-18, ADAM10 expression is upregulated, leading to increased secretion of HER ligands. This subsequently promotes HER1/HER2/HER3 heterodimerisation and activation of downstream PI3K/AKT and ERK signalling pathways, ultimately inducing trastuzumab resistance [33].

##### ADAM10/γ-Secretase-Mediated Cleavage and Nuclear Translocation of p75HER2

At the transcriptional and post-translational levels, studies have shown that HER2 regulates tumour cell proliferation and drug resistance via non-classical pathways.

Interestingly, while trastuzumab does not inhibit HER2 homodimerization or phosphorylation in CHO cells stably expressing HER2, it still suppresses their proliferation [34,35], suggesting trastuzumab may inhibit tumour growth by targeting non-canonical HER2 pathways. 

Research by Nami et al. revealed that HER2 regulates transmembrane proteolysis via a non-canonical pathway. In this pathway, HER2 is initially cleaved by the metalloproteinase ADAM10 to generate a released extracellular domain and p95 HER2 (containing a transmembrane domain and an intracellular domain). Following cleavage of p95 HER2 by the transmembrane protease γ-secretase, a soluble intracellular domain, p75 HER2, bearing a nuclear localisation signal is generated. p75HER2 undergoes phosphorylation and translocates to the nucleus, where it continues to exert its pro-proliferative function. Their work further revealed that trastuzumab targets this non-canonical HER2 pathway by inhibiting ADAM10 and γ-secretase-mediated proteolytic cleavage of HER2. Trastuzumab suppresses HER2-induced cell proliferation but does not inhibit p75HER2-induced proliferation [36]. The aforementioned studies indicate that HER2-induced proliferation in tumour cells exhibits resistance to trastuzumab. However, this resistance can be overcome by employing ADAM10 and γ-secretase inhibitors. 

##### DPAGT1 Mediates Trastuzumab Resistance by N-Glycosylating ADAM10

Research by Yang et al. elucidated the molecular mechanism whereby the endoplasmic reticulum integration membrane enzyme DPAGT1 promotes HER2 cleavage and trastuzumab resistance by regulating the N-glycosylation of ADAM10. DPAGT1 mediates glycosylation modification of ADAM10 at the N267 site, thereby protecting ADAM10 from endoplasmic reticulum-associated degradation (ERAD). This ensures its stability, maturation, and membrane-localised activity, ultimately leading to resistance. Trastuzumab treatment induces DPAGT1 translocation from the plasma membrane to the endoplasmic reticulum via vesicle-mediated retrograde transport, further enhancing ADAM10 glycosylation [37]. 

##### Treatment Strategies for p95HER2-Mediated Resistance

These findings not only deepen our understanding of the mechanisms underlying HER2 heterogeneous resistance, but also provide a theoretical basis for the development of combination therapeutic strategies targeting ADAM10, γ-secretase or DPAGT1. They suggest that future efforts should integrate epigenetic modifications, proteasome activity and cross-regulation of signalling pathways to overcome resistance to HER2-targeted therapy and improve the prognosis of breast cancer patients.

Several therapeutic strategies targeting p95HER2-mediated resistance have demonstrated potential in preclinical and clinical studies. Firstly, as p95HER2 lacks the extracellular domain required for binding to trastuzumab but retains a complete intracellular kinase domain [31,38], irreversible pan-HER tyrosine kinase inhibitors (such as neratinib and pyrotinib) are able to effectively inhibit its kinase activity. Preclinical studies have shown that neratinib significantly inhibits tumour growth in cells overexpressing p95HER2 and in xenograft models [39]. Secondly, bispecific antibodies (such as zanidatamab) can simultaneously bind to multiple epitopes on HER2; by inducing receptor aggregation and internalisation, they partially overcome the resistance caused by the absence of the extracellular domain. A related Phase Ib/II clinical trial is currently evaluating its efficacy in patients with HER2-positive breast cancer [40]. In clinical practice, for patients who have progressed following treatment with trastuzumab and in whom high p95HER2 expression has been detected, switching to an irreversible TKI or participating in a clinical trial of a bispecific antibody may be considered.

### 2.2 HER2-Mediated Drug Resistance through Interactions with Other Molecules

#### 2.2.1 HER Family-Mediated Bypass

HER3, as the primary adaptor protein in the PI3K/AKT pathway, has its ligand NRG1 (neuregulin 1) capable of restarting downstream signalling by inducing HER2-HER3 heterodimeric complexes [41]. Research by Yang et al. revealed that in the absence of NRG1 stimulation, trastuzumab significantly downregulated p-HER2 (phosphorylated human epidermal growth factor receptor 2) expression and induced increased early apoptosis in BT474 cells (trastuzumab-sensitive). Following NRG1 stimulation, however, trastuzumab’s inhibitory effect was abolished. They observed increased p-HER3 (phosphorylated human epidermal growth factor receptor 3) expression following NRG1 stimulation, suggesting that NRG1-induced p-HER3 upregulation may counteract trastuzumab-induced p-HER2 downregulation, potentially leading to trastuzumab resistance [42]. The NRG1/HER3 axis is crucial for proliferation in HER2-overexpressing cells [43]. A study by Ebbing et al. demonstrated that the metalloproteinase ADAM10 can induce trastuzumab resistance by releasing NRG1 from the cell membrane, thereby activating HER3 and downstream signalling pathways [44]. These studies collectively indicate a strong association between NRG1/HER3 and trastuzumab resistance, suggesting that anti-HER3 therapy may reverse such resistance. Furthermore, Udagawa et al. employed lentiviral transfection to fuse the NRG1 gene with known fusion partners (such as CD74, SLC3A2, etc.) into lung cancer cell lines via lentiviral transduction. This approach preserved the EGF-like domain of NRG1, ensuring its binding to HER receptors and activation of downstream signalling pathways. The resulting NRG1 fusion-positive cancer models revealed the roles of HER4 and EGFR (HER1) in promoting downstream signalling and cellular growth within these tumours [45]. The aforementioned studies demonstrate that HER1, HER3, and HER4 within the HER family can all activate downstream signalling via bypass mechanisms, thereby promoting tumour growth and the development of drug resistance.

Momeny et al. discovered that in HER2-positive breast cancer, the mechanism of TKI resistance primarily involves a non-genetic resistance pathway driven by a DUSP6 (Bispecific phosphatase 6)–HER3 positive feedback loop. During the initial phase of drug treatment, tumour cells enter a dormant state (DTP), where expression of DUSP6 is suppressed. However, in the recurrent proliferation phase (DTEP), DUSP6 expression is significantly upregulated. This upregulation promotes HER3 expression through transcriptional regulation, thereby activating the neuregulin (NRG)–HER3 signalling axis and mediating TKI resistance. Furthermore, DUSP6 inhibits apoptosis via ERK-independent pathways, promoting cellular survival. In acquired-resistance cells, TKIs fail to effectively inhibit the MEK–ERK–DUSP6 pathway, leading to sustained HER3 overexpression and maintenance of the resistant phenotype. Inhibiting DUSP6 disrupts this circuit, reduces HER3 levels, and restores TKI sensitivity with superior efficacy compared to AKT inhibition strategies [46].

#### 2.2.2 Non-HER Membrane Proteins

##### C-MET: Activates the PI3K/AKT Pathway and Mediates Resistance to Trastuzumab

Numerous studies have demonstrated that the co-expression or interaction of certain proteins with HER2 can similarly promote sustained activation of downstream signalling pathways, thereby driving tumour progression and drug resistance. 

The hepatocyte growth factor receptor (c-MET) frequently co-expresses with HER2 in HER2-positive breast cancer and promotes resistance to trastuzumab through sustained activation of the PI3K/Akt/mTOR pathway [47]. Research by Shattuck et al. revealed that c-MET activation protects tumour cells from trastuzumab-induced cell death. Furthermore, HER2-overexpressing breast cancer cells exhibit upregulation of c-MET expression following trastuzumab treatment, thereby promoting resistance [48].

With regard to clinical patient data, research indicates that c-MET amplification correlates with trastuzumab treatment failure and shorter progression-free survival [49], whilst high expression of phosphorylated c-MET is associated with poor prognosis in breast cancer [50]. 

In addition, a large body of preclinical research has demonstrated the interaction between the relevant molecules and HER2.

##### TRAF4 and CMTM6: Inhibiting the Ubiquitination and Degradation of HER2, thereby Stabilising the HER2 Protein

Tumour Necrosis Factor Receptor-Associated Protein 4 (TRAF4) is an oncogenic adaptor protein, with established evidence demonstrating its pivotal role in cancer progression, metastasis, and the development of drug resistance [51,52,53]. Research by Gu et al. revealed the molecular mechanism by which TRAF4 drives trastuzumab resistance through regulating the HER2 ubiquitination and degradation pathway mediated by SMURF2 (SMAD-specific E3 ubiquitin ligase protein, an E3 ligase targeting HER2 and promoting its ubiquitination and degradation). In HER2-positive breast cancer, TRAF4 overexpression maintains HER2 signalling through two mechanisms: firstly, TRAF4 directly degrades SMURF2 as an E3 ubiquitin ligase, thereby reducing SMURF2’s inhibitory effect on HER2; secondly, TRAF4 competitively binds to the intracellular domain of HER2, blocking SMURF2-HER2 interaction and further stabilising HER2 protein levels. This dual regulation leads to sustained activation of HER2 and its downstream AKT/mTOR signalling pathways, undermining trastuzumab-induced HER2 degradation and signal inhibition. Moreover, trastuzumab treatment feedback-upregulates TRAF4 expression, establishing a resistance cycle. Knockdown or knockout of TRAF4 restores SMURF2 function, promotes HER2 ubiquitination and degradation, and inhibits AKT/mTOR pathway activity, thereby significantly enhancing tumour cell sensitivity to trastuzumab [54]. This mechanism provides a novel target for overcoming resistance to targeted therapies in HER2-positive breast cancer. Research by Xing et al. has revealed that CMTM6 (CKLF-like MARVEL transmembrane domain-containing protein 6) directly interacts with HER2, enhancing HER2-related signalling pathways. Furthermore, by limiting HER2 ubiquitination, it stabilises the HER2 protein within tumour cells [55].

##### MUC4: Promotes HER2/HER3 Dimerisation and Masks Antigenic Epitopes

Mucin 4 (MUC4) promotes the dimerisation of receptors such as HER2 and HER3, thereby activating downstream signalling pathways (e.g., MAPK/ERK) and inhibiting apoptosis. Furthermore, MUC4 overexpression masks tumour cell surface antigens, diminishing immune recognition, and promotes cell detachment from the primary site through anti-adhesive properties. This synergises with HER2 signalling to jointly drive breast cancer cell invasion, migration, anti-apoptotic behaviour, and metastatic potential [56]. Regarding the molecular mechanisms regulating MUC4, Perez et al. demonstrated that in rat mammary carcinoma cells, the transcription factor PEA3 directly binds the MUC4 promoter region, mediating MUC4 transcription via ERK and SAPK/JNK signalling pathways [57].

##### GRB7-Associated Signalling Axes (FAK/GRB7, circCDYL2, TCF12/Notch1): Activation of the PI3K/AKT, RAS/ERK and Wnt/β-Catenin Pathways

Focal adhesion kinase (FAK) phosphorylates GRB7 on its tyrosine residues. The resulting FAK/GRB7 complex regulates the phosphorylation of AKT and ERK1/2, thereby promoting cancer cell migration and proliferation [58]. Ling et al. discovered that the circular RNA circCDYL2 stabilises GRB7 by inhibiting ubiquitin-mediated degradation and enhances its interaction with FAK. This drives cell proliferation by activating the PI3K/AKT and RAS/ERK1/2 signalling pathways, thereby diminishing the therapeutic efficacy of trastuzumab [59]. Wang et al. demonstrated that transcription factor 12 (TCF12) binds to the promoter region of growth factor receptor-binding protein 7 (GRB7), thereby promoting its transcriptional expression. Their work further demonstrated that GRB7 binds to the Notch1 receptor on the cell membrane, increasing the accumulation of its ligand Jagged1. This activates the Wnt/β-catenin signalling pathway and other pathways (such as PI3K/AKT and MAPK/ERK), thereby promoting the progression of epithelial-mesenchymal transition (EMT) in HER2-positive breast cancer [60].

#### 2.2.3 Chaperones and Stability Regulators

Research has confirmed that HSP90 (heat shock protein 90) has over 200 client proteins, including transcription factors and tyrosine kinases [61,62]. HSP90 plays a crucial role in regulating the activation and dimerisation of receptor tyrosine kinases (HER2, HER3) [63]. By binding to HER2, HSP90 maintains its conformational stability and inhibits its ubiquitination and degradation. Numerous studies demonstrate that HSP90 inhibitors (such as HVH-2930, NVP-AUY922, and DCZ3112) exhibit potent efficacy against HER2-positive breast cancer cells resistant to trastuzumab [39,64,65]. These findings indicate a close association between HSP90 and the development of trastuzumab resistance.

Itah et al. discovered that Jun N-terminal kinase (JNK) negatively regulates HER2-driven breast cancer progression by suppressing the expression of integrin α6β4 and its interaction with HER2. In HER2-positive breast cancer, deletions or loss-of-function mutations in the MAP2K4 and MAP2K7 genes (encoding JNK upstream kinases) reduce JNK activity, thereby releasing transcriptional repression of integrin α6β4. Upon binding to HER2, integrin α6β4 significantly enhances HER2 downstream signalling (e.g., ERK and AKT pathways). *In vivo* experiments further confirmed that JNK pathway deficiency (including MAP2K4 or MAP2K7 knockout) accelerates HER2-driven mouse breast cancer development [66]. This mechanism demonstrates that the JNK pathway functions as a tumour suppressor by limiting HER2 signal amplification to inhibit breast cancer progression. Its functional loss is closely associated with drug resistance and malignant progression in HER2-positive breast cancer.

**Figure 1:**
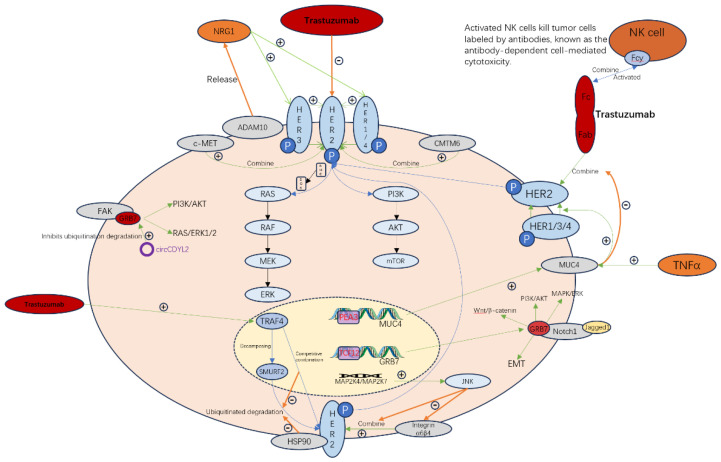
HER2-Mediated Drug Resistance Through Interactions with Other Molecules. This figure summarises the molecular network underlying resistance to anti-HER2 therapy in HER2-positive breast cancer, arising from interactions between HER2 and non-HER family membrane proteins, adaptor proteins, and chaperone proteins. This primarily includes: (**1**) NRG1 activates the HER2-HER3 heterodimer and remodels HER3 signalling via ADAM10; (**2**) The DUSP6-HER3 positive feedback loop drives TKI resistance; (**3**) c-MET, TRAF4 and CMTM6 inhibit HER2 ubiquitination and degradation, stabilising the HER2 protein; (**4**) HSP90 maintains the structural stability of HER2; (**5**) Inactivation of the JNK pathway lifts transcriptional repression of integrin α6β4, amplifying HER2 signalling; (**6**) MUC4 masks HER2 epitopes and promotes dimerisation; (**7**) GRB7 and circCDYL2 activate the PI3K/AKT and Wnt/β-catenin pathways, inducing EMT and resistance. The figure also identifies potential intervention targets (such as HER3, c-MET, TRAF4, HSP90, DUSP6, etc.), providing a theoretical basis for combination therapy.

NRG1 circumvents HER2 inhibition by activating the HER2-HER3 heterodimer and reshaping HER3 signalling via ADAM10; the DUSP6-HER3 positive feedback loop, meanwhile, drives the non-genetic dormancy-relapse process in TKI resistance. Furthermore, molecules such as c-MET, TRAF4 and CMTM6 promote resistance by inhibiting ubiquitin-mediated degradation, stabilising HER2 protein and enhancing downstream AKT/ERK signalling, respectively; whilst HSP90 maintains HER2 conformation stability, and the loss of the JNK pathway lifts transcriptional repression of integrin α6β4, thereby amplifying HER2 signalling. MUC4 masks HER2 epitopes and promotes receptor dimerisation, whilst GRB7 and circCDYL2 drive epithelial-mesenchymal transition and resistance by activating the PI3K/AKT and Wnt/β-catenin pathways. These studies reveal the complex bypass activation, protein stability regulation and signalling network reprogramming of HER2, suggesting that combined targeting of key nodes such as HER3, c-MET, TRAF4, HSP90 or DUSP6 holds promise for reversing drug resistance, thereby providing a theoretical basis for the development of precision intervention strategies based on signalling interaction networks (Fig. 1).

### 2.3 Drug Resistance Mediated by the HER2 Downstream Signalling Pathway

#### 2.3.1 MAPK and PI3K Signalling Pathways

##### PTEN Loss and PIK3CA Mutations Drive PI3K/AKT Activation

PTEN is a PIP3-specific phosphatase that dephosphorylates PIP3 molecules into PIP2, thereby preventing AKT recruitment to the cell membrane and inhibiting the PI3K/AKT/mTOR signalling pathway. PTEN loss (observed in 40–50% of HER2-positive breast cancers) results in sustained activation of the PI3K/AKT/mTOR signalling pathway, promoting tumour proliferation and progression [67,68]. PI3K comprises a regulatory subunit (p85) and a catalytic subunit (p110), whilst PIK3CA is a frequently mutated gene encoding the p110α catalytic subunit of the PI3K pathway. PIK3CA mutations (observed in 30–40% of breast cancer patients) similarly activate the PI3K/AKT pathway. Both bypass HER2-dependent growth signalling, thereby promoting resistance [5]. 

##### PI3K PROTACs Overcome Lapatinib Resistance

Zhang et al. discovered that synthetic PI3K PROTACs (proteolysis-targeting chimeras) can inhibit breast cancer cell proliferation and induce G1 cell cycle arrest by specifically degrading PI3K-p110α protein. In lapatinib-resistant HER2-positive breast cancer models (including PIK3CA-mutant cell lines, xenografts, and patient-derived organoids), this molecule effectively restored tumour cell sensitivity to lapatinib, demonstrating superior efficacy compared to the clinically used PI3K-p110α kinase inhibitor alpelisib. Research indicates that PI3K PROTACs can reverse lapatinib resistance by degrading PI3K-p110α to block excessive activation of the PI3K-AKT signalling pathway [69].

##### IGF-1R and Cullin7 Regulate Trastuzumab Sensitivity via IRS-1

The insulin-like growth factor 1 receptor (IGF-1R) promotes tyrosine phosphorylation of insulin receptor substrate-1 (IRS-1), hyperactivating the activity of the PI3K/Akt/mTOR pathway and thereby diminishing the efficacy of trastuzumab. Qiu et al. demonstrated that knocking down Cullin7 in trastuzumab-resistant HER2-positive breast cancer cells reduces degradation of serine-phosphorylated IRS-1. This blocks tyrosine phosphorylation, prevents IRS-1 binding to insulin receptor (IR), attenuates PI3K/AKT pathway activation, and partially restores trastuzumab sensitivity [70]. These studies suggest Cullin7 may serve as a biomarker for improving treatment strategies against HER2-targeted therapy resistance.

##### CD147 Induces EMT and Drug Resistance via MAPK/ERK

CD147 is a transmembrane glycoprotein highly expressed in breast cancer, which promotes tumour migration and invasion by inducing epithelial-mesenchymal transition (EMT) through activation of the MAPK/ERK signalling pathway. Experiments by Li et al. demonstrated that in breast cancer cells overexpressing CD147, the expression of Snail1, Vimentin, and MMP-9 was significantly upregulated, whilst E-cadherin expression was downregulated. Concurrently, the phosphorylation levels of MEK and ERK increased, indicating activation of the MAPK/ERK pathway. Conversely, knocking down CD147 reversed these effects, inhibiting changes in EMT markers and reducing pathway activity [71]. These findings indicate that CD147 drives the EMT process by regulating the MAPK/ERK pathway, thereby mediating drug resistance.

##### The TMBIM6–miR-181a–MAPK/ERK Axis in EMT and Invasion

Research has revealed that TMBIM6 (transmembrane Bax inhibitor motif-containing protein 6) is highly expressed in breast cancer. It activates the MAPK/ERK signalling pathway by upregulating miR-181a (a microRNA), thereby inducing epithelial-mesenchymal transition (EMT) and cellular invasion. Experiments by Shin et al. demonstrated that knocking down TMBIM6 significantly inhibited breast cancer cell proliferation, migration, and expression of EMT markers, while concurrently reducing ERK phosphorylation levels and miR-181a expression. Conversely, TMBIM6 overexpression drives EMT by enhancing MAPK/ERK pathway activity, upregulating Snail-1 and Snail-2 expression, suppressing E-cadherin, and promoting mesenchymal marker expression. Furthermore, TMBIM6 increases MMP-9 secretion and activity via a miR-181a-dependent mechanism, thereby enhancing invasive capacity. Inhibition of the MAPK/ERK pathway reverses these effects [72]. These findings indicate that the TMBIM6-miR-181a-MAPK/ERK axis plays a pivotal role in breast cancer migration and invasion by regulating EMT-associated transcription factors and matrix metalloproteinases (MMPs).

##### POU4F1 Activates ERK1/2 to Confer Trastuzumab Resistance

POU4F1, a POU domain transcription factor, plays roles in cancer regulation [73,74,75]. Wu et al. discovered that POU4F1 mediates trastuzumab resistance in HER2-positive breast cancer by activating the ERK1/2 signalling pathway. In trastuzumab-resistant breast cancer clinical samples and cell lines, POU4F1 expression was significantly upregulated and closely associated with poor patient prognosis and tumour recurrence. *In vitro* and *in vivo* experiments demonstrated that POU4F1 knockdown suppressed proliferation, migration, and invasion in trastuzumab-resistant cells. This effect was mediated by blocking the ERK signalling pathway through reduced phosphorylation of MEK1/2 and ERK1/2, thereby restoring cellular sensitivity to trastuzumab. Furthermore, activation of ERK1/2 reverses the inhibitory effect of POU4F1 knockdown on the resistant phenotype, confirming the central role of the ERK pathway in this mechanism [76]. These findings suggest that targeting the POU4F1-ERK1/2 axis may represent a novel strategy to overcome trastuzumab resistance.

##### MYO6 Enhances MAPK/ERK Signaling to Promote Drug Resistance

Extensive research has demonstrated that myosin VI (MYO6) functions as an oncogene associated with tumour progression in several cancers [77,78]. Research by Zhan et al. revealed that MYO6 participates in the MAPK/ERK pathway and demonstrated that it enhances breast cancer proliferation, migration, and invasion by increasing the expression of phosphorylated ERK1/2, thereby promoting drug resistance [79]. This suggests that MYO6 may represent a novel potential therapeutic and prognostic target for breast cancer patients.

#### 2.3.2 Other Downstream Signalling Pathways

Extensive research has explored the MAPK and PI3K signalling pathways in drug-resistant breast cancer to elucidate mechanisms associated with resistance and identify novel therapeutic targets. Beyond these classical pathways, studies have uncovered additional mechanisms linked to breast cancer drug resistance. 

##### NCAPG/SRC/STAT3 Signalling Axis

Jiang et al. observed that non-structural maintenance of chromosomes condensin I complex subunit G (NCAPG) is highly expressed in HER2-positive breast cancer, mediating trastuzumab resistance by activating the SRC/STAT3 signalling pathway. Experiments demonstrated that NCAPG was significantly upregulated in trastuzumab-resistant breast cancer tissues and cell lines, with its overexpression correlated with patient recurrence and poor prognosis. *In vivo* and *in vitro* studies showed that knocking down NCAPG inhibited tumour proliferation and enhanced trastuzumab-induced apoptosis, thereby reversing resistance; conversely, overexpressing NCAPG promoted cell proliferation and suppressed apoptosis. Mechanistically, NCAPG enhances Tyr416 phosphorylation of SRC, thereby activating downstream STAT3 nuclear translocation and transcriptional activity, which upregulates target gene expression including Cyclin D1 (proliferation-associated) and BCL2 (anti-apoptotic) [80]. Thus, NCAPG drives trastuzumab resistance via the SRC/STAT3 signalling axis, suggesting its potential value as a prognostic marker and therapeutic target.

##### PHF8/IL-6/STAT3 Signalling Axis

Research by Liu et al. indicates that the histone demethylase PHF8 activates the IL-6/STAT3 signalling axis by regulating cytokine secretion (including IL-6), thereby inhibiting apoptosis and enhancing tumour cell resistance to trastuzumab and lapatinib [30]. 

##### TAM-Derived IL-8/CXCR1/2/SRC/STAT3/ERK1/2 Axis

Research by Ahmed et al. revealed that in HER2-positive locally advanced breast cancer (LABC), tumour-associated macrophages (TAMs) activate the SRC/STAT3/ERK1/2 signalling pathway through substantial IL-8 secretion, thereby mediating lapatinib resistance. IL-8 binds its receptor CXCR1/2, triggering SRC kinase activation which subsequently phosphorylates EGFR (Tyr845) and HER2, thereby activating STAT3 and ERK1/2. The activation of these signalling pathways counteracts lapatinib’s inhibition of EGFR/HER2 tyrosine kinases, enabling cancer cells to maintain proliferation, invasiveness, and stem-like properties in the presence of lapatinib [81].

##### Hippo/YAP Signalling Pathway

The Hippo pathway, a conserved kinase cascade, regulates cell proliferation and apoptosis by controlling YAP/TAZ (Yes-associated protein/transcriptional coactivator with PDZ-binding motif) activity. Dysregulation of this pathway is linked to cancer [82]. González-Alonso et al. discovered that abnormal activation of key effector molecules YAP1 and TEAD2 (TEA domain transcription factor 2) within the Hippo pathway may induce acquired resistance to trastuzumab. They observed that in resistant cell lines, YAP1 undergoes dephosphorylation and nuclear translocation, where it synergises with the transcription factor TEAD2 to upregulate downstream pro-proliferative genes (such as AREG, CTGF, and VEGFA), thereby driving cell survival, migration, and epithelial-mesenchymal transition (EMT). Multi-omics analysis revealed enhanced Hippo pathway activity in resistant cells, manifested by reduced YAP1 phosphorylation and TEAD2 overexpression. Furthermore, nuclear accumulation of YAP1/TAZ in clinical samples was significantly correlated with poorer patient survival. *In vitro* and *in vivo* experiments confirmed that silencing or pharmacologically inhibiting the YAP1/TEAD complex restored trastuzumab sensitivity, with combined therapy significantly suppressing tumour growth and inducing apoptosis [83]. Zou et al. further revealed that the SRC kinase promotes tyrosine phosphorylation of YAP1, facilitating its binding with the transcription factor TFAP2A to form the YAP1/TEAD-TFAP2A (YTT) complex. This complex enhances cancer stem cell properties and activates pro-survival signalling pathways by synergistically regulating the transcription of genes such as EGFR, HER2, H19, and CTGF, thereby driving drug resistance. Experiments demonstrated that inhibiting SRC activity or disrupting the YTT complex significantly restored cellular sensitivity to trastuzumab [84]. The tyrosine phosphorylation site of YAP1 mediated by SRC kinase differs from the classical Hippo pathway. In the classical pathway, YAP1 phosphorylation leads to degradation of YAP/TAZ, thereby inactivating the Hippo pathway. Conversely, SRC kinase-mediated tyrosine phosphorylation of YAP1 activates the Hippo pathway. The aforementioned studies indicate that targeting Hippo pathway effectors and YTT complex-associated transcriptional regulation in combination with anti-HER2 therapy may represent a novel strategy to overcome trastuzumab resistance.

##### PRKACA/BAD Anti-Apoptotic Pathway

Additionally, Moody et al. discovered that the survival kinase PRKACA inactivates the pro-apoptotic protein BAD through phosphorylation, thereby releasing BAD’s inhibitory effect on the anti-apoptotic proteins BCL-XL/BCL-2. This restores the cell survival signal, subsequently mediating HER2-positive breast cancer cell resistance to trastuzumab and lapatinib [85]. This mechanism operates independently of classical HER2-downstream signalling pathways such as MAPK or PI3K/AKT, instead achieving its effect through direct regulation of the apoptotic pathway.

**Figure 2:**
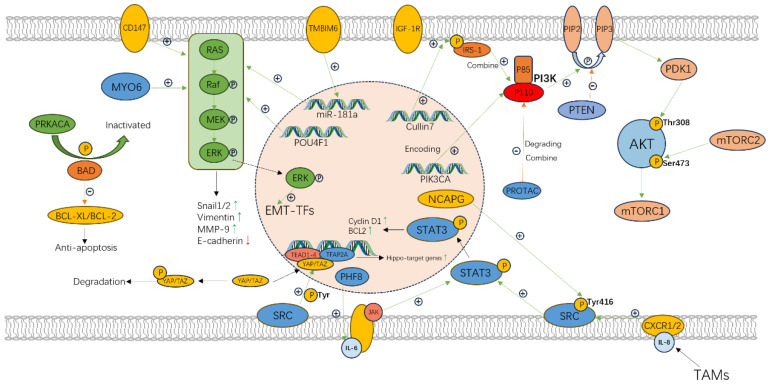
Drug resistance mediated by the HER2 downstream signalling pathway. This figure illustrates how the abnormal activation of classical and non-classical downstream HER2 signalling pathways leads to resistance to anti-HER2 therapy. These primarily include: (**1**) the PI3K/AKT/mTOR pathway: PTEN loss or PIK3CA mutations result in sustained activation of the pathway; a PI3K PROTAC that degrades p110α can reverse resistance; (**2**) MAPK/ERK pathway: molecules such as CD147, TMBIM6, MYO6 and POU4F1 promote EMT, migration and resistance by activating this pathway; (**3**) SRC/STAT3 pathway: NCAPG, PHF8 and TAMs secrete IL-8 and other factors to activate this pathway, upregulating Cyclin D1 and BCL2; (**4**) Hippo/YAP pathway: YAP1 is dephosphorylated and translocates to the nucleus, where it forms a complex with TEAD2 or TFAP2A to upregulate pro-proliferative genes; SRC kinase mediates the tyrosine phosphorylation of YAP1 to activate this pathway; (**5**) Apoptosis regulation: PRKACA phosphorylates BAD, thereby lifting the inhibition of BCL-XL/BCL-2 and promoting anti-apoptotic effects. The figure highlights the cross-talk between multiple pathways, suggesting that combined targeting (e.g., PI3K + MAPK, STAT3 + YAP1 inhibitors) is a potential strategy for overcoming heterogeneous resistance.

The above studies indicate that, in addition to the classical PI3K/AKT and MAPK/ERK pathways, the SRC/STAT3, Hippo/YAP and apoptosis regulatory networks are all involved in the development of drug resistance. Targeting these key nodes—such as developing PI3K-p110α PROTAC degraders, co-inhibiting the YAP1/TEAD complex, blocking SRC kinase activity, or targeting the IL-8/CXCR1/2 axis—holds promise for reversing drug resistance; Furthermore, given the extensive cross-talk between signalling pathways, multi-target combined interventions (such as simultaneously targeting the PI3K and MAPK pathways, or combining STAT3 and YAP1 inhibitors) may represent an effective strategy for overcoming heterogeneous resistance, offering a new direction for precision therapy in HER2-positive breast cancer (Fig. 2).

### 2.4 Payload- and Trafficking-Dependent Resistance to ADCs

ADCs combine the targeting capabilities of monoclonal antibodies with cytotoxic payloads to exert their anti-tumour effects. Any abnormality in this process may lead to drug resistance. In addition to resistance caused by alterations in HER2 expression and recognition defects, this primarily includes impaired internalisation and transport, as well as lysosomal dysfunction.

#### 2.4.1 Impaired Cellular Internalisation and Transport

After binding to its target molecule, an ADC must enter the tumour cell via endocytosis in order to exert its effect. The inability of HER2 to undergo endocytosis is one of the primary mechanisms of ADC resistance. Research by Sung et al. indicates that T-DM1-resistant NCI-N87 cells upregulate caveolin-1 (CAV1) and caveolin-2, which are essential for caveolae formation. This may promote drug uptake whilst limiting lysosomal degradation, ultimately reducing the efficacy of T-DM1 [86]. Furthermore, it has been demonstrated in recent years that SNX10 deficiency alters HER2 intracellular trafficking. The mechanism involves a decrease in the activity of the transcription factor STAT1 following SNX10 loss, which subsequently downregulates the transcription and expression of RAB11A. This impairs the ability of HER2 to recycle from endosomes to the plasma membrane, whilst simultaneously promoting the transport of HER2 to lysosomes for degradation; the combined effects of impaired HER2 recycling and enhanced lysosomal degradation reduce HER2 levels on the cell membrane, thereby leading to reduced sensitivity of HER2-positive breast cancer cells to T-DM1 and T-DXd [87].

#### 2.4.2 Lysosomal Dysfunction

Following internalisation, ADCs must be transported to the lysosomes for degradation and payload release; impaired lysosomal function hinders this process. Research by Wang et al. revealed that a significant reduction in lysosomal V-ATPase activity leads to impaired lysosomal acidification, thereby reducing the generation and accumulation of active metabolites of T-DM1. This decrease in metabolite levels prevents T-DM1 from effectively inhibiting microtubule polymerisation, consequently leading to drug resistance. It is worth noting that resistant cells remain sensitive to trastuzumab and free DM1, whilst the use of HER2-targeted ADCs containing cleavable linkers can effectively overcome this resistance [88]. Kevin J Hamblett and colleagues found that non-cleavable linker-containing metaxin-type antibody-drug conjugates (such as T-DM1), after being cleaved into amino acid-linker-payload (e.g., lysine-MCC-DM1) within lysosomes, must rely on the lysosomal membrane transporter SLC46A3 to be transported from the lysosomes into the cytoplasm in order to exert their microtubule-inhibiting effect; when SLC46A3 expression is downregulated or its function is impaired, the active metabolite accumulates within the lysosome and is unable to enter the cytoplasm, leading to a significant reduction in the ADC’s antitumour activity and, consequently, the development of resistance. This mechanism is independent of cell surface targets or linker structure, but is specific to metaxamine-based payloads, whereas ADCs utilising cleavable linkers remain unaffected [89].

#### 2.4.3 Payload-Related Resistance

The payload is the core active molecule responsible for ADC-mediated tumour cell killing; resistant cells can attenuate the payload’s effect through various mechanisms. One key factor involves increased drug efflux, whereby resistant cells upregulate ATP-binding cassette (ABC) transporters (such as ABCC1, ABCG2 and MDR1) to actively pump the payload out of the cell, thereby reducing intracellular drug concentrations. This type of efflux pump-mediated resistance is independent of HER2 expression levels or internalisation processes; rather, it directly affects the intracellular accumulation of the payload, constituting a non-target-dependent, payload-specific resistance mechanism [90]. In addition, loss of SLX4 function is a key factor; as a regulator of DNA repair pathways, loss-of-function mutations in SLX4 are one of the indicators of T-DXd resistance [91].

#### 2.4.4 Tumour Microenvironment Factors

The tumour microenvironment also plays a significant role in ADC resistance. Resistance mechanisms include alterations in the immunosuppressive state of the microenvironment and barriers to drug penetration. Studies have found that p95HER2 can drive tumour immune evasion by promoting PD-L1 expression and secreting immunosuppressive mediators such as IL-6, thereby undermining the therapeutic efficacy of T-DXd, which relies on immunogenic cell death to achieve its therapeutic effect [31,92]. Zou et al. identified crVDAC3 as a key gene conferring T-DXd resistance in HER2-low breast cancer through shRNA library screening. In HER2-low breast cancer cells, crVDAC3 specifically binds to HSPB1 (Heat Shock Protein Family B (Small) Member 1) and inhibits its ubiquitination and degradation, leading to intracellular accumulation of HSPB1. Elevated HSPB1 protein levels suppress ferroptosis by inhibiting excessive reactive oxygen species (ROS) activity and accumulation of unstable iron pools, ultimately mediating T-DXd resistance. Paritaprevir binds to crVDAC3, blocking its interaction with HSPB1 and promoting HSPB1 ubiquitination and degradation, thereby reversing this resistance mechanism [93].

## 3 Mechanisms of Drug Resistance Mediated by the Tumour Microenvironment

### 3.1 Immune-Related Mechanisms

Immunosuppressive components within the tumour microenvironment (TME), particularly tumour-associated macrophages (TAMs), the RNA editing enzyme ADAR1, and the non-classical MHC molecule HLA-G, collectively promote resistance to anti-HER2 therapy by attenuating antibody-dependent cell-mediated cytotoxicity (ADCC), inhibiting type I interferon (IFN) signalling, and blocking natural killer (NK) cell function.

#### 3.1.1 Inhibition of ADCC by Tumour-Associated Macrophages

M2-type TAMs constitute the primary immunosuppressive cell population within the tumour microenvironment (TME). They suppress the activation of T cells and NK cells by secreting IL-10 and TGF-β, and by expressing negative co-stimulatory molecules such as PD-L1 and B7-H4 [94]. In treatment with anti-HER2 monoclonal antibodies (such as trastuzumab), ADCC is one of the key anti-tumour mechanisms. TAMs secrete CSF-1 to maintain their own survival and further recruit more M2 macrophages via the CSF-1/CSF-1R axis [95]. TAMs can also express high levels of CD47, which, upon binding to SIRPα on the surface of phagocytes, negatively regulates phagocytosis, thereby reducing the clearance of antibody-labelled tumour cells [96]. Furthermore, TAMs deplete arginine and tryptophan in the microenvironment via arginase (ARG1) and indole-2,3-dioxygenase (IDO), leading to T-cell metabolic failure [97].

Small-molecule inhibitors targeting CSF-1/CSF-1R (such as pexidartinib) can reduce TAM infiltration and restore the ADCC activity of trastuzumab in preclinical models [98]. Antibodies that block the CD47-SIRPα interaction (such as magrolimab) have also demonstrated potential for synergy with anti-HER2 therapy in early clinical trials [99].

#### 3.1.2 Inactivation of the Type I Interferon Pathway Mediated by ADAR1

The long non-coding RNA LINC00624 binds to the ADAR1 protein via its double-stranded RNA-like structure, thereby inhibiting the β-TrCP-mediated ubiquitination and degradation of ADAR1, thus enhancing the stability of ADAR1. As an RNA editor, ADAR1 disrupts the secondary structure of intracellular double-stranded RNA through A-to-I editing, thereby inhibiting the activation of innate immune sensors such as RIG-I and MDA5. This leads to the suppression of the type I IFN signalling pathway and the expression of its downstream interferon-stimulated genes (ISGs), ultimately resulting in reduced antigen presentation by MHC class I molecules and decreased CD8^+^ T-cell infiltration, creating a ‘cold’ TME and reducing the efficacy of anti-HER2 monoclonal antibodies and TKIs [100,101].

The use of antisense oligonucleotides (ASOs) targeting LINC00624 restores ADAR1 degradation, reactivates the IFN signalling pathway, and enhances anti-tumour immunity, providing a new strategy for overcoming drug resistance [102].

#### 3.1.3 NK Cell Inhibition Mediated by the HLA-G/KIR2DL4 Axis

The non-classical MHC molecule HLA-G is upregulated in HER2-positive breast cancer cells. Upon binding to the inhibitory receptor KIR2DL4 on the surface of NK cells, HLA-G directly inhibits NK cell-mediated ADCC effects. Trastuzumab treatment induces tumour cells to secrete TGF-β whilst simultaneously stimulating NK cells to release IFN-γ, forming a positive feedback loop: TGF-β further upregulates HLA-G and PD-L1 on the surface of tumour cells, whilst IFN-γ enhances the expression of KIR2DL4 and PD-1 on NK cells via the JAK2/STAT1 pathway, thereby doubly inhibiting NK function. Blocking the HLA-G/KIR2DL4 interaction or combining treatment with PD-1/PD-L1 immune checkpoint inhibitors can restore NK cell ADCC activity and reverse drug resistance [103].

#### 3.1.4 Combination Immunotherapy Strategies

Given the aforementioned immune evasion mechanisms, combination immunotherapy holds promise for overcoming resistance to anti-HER2 therapy. Several preclinical and early-phase clinical studies are currently exploring the following regimens:

**Anti-HER2 therapy + immune checkpoint inhibitors:** Although HER2-positive breast cancer is typically classified as a ‘cold’ tumour, with limited efficacy when PD-1/PD-L1 inhibitors are used alone, combination with trastuzumab or T-DXd can produce synergistic effects. For example, the KATE2 trial evaluated the efficacy of T-DM1 combined with atezolizumab (anti-PD-L1) in patients with advanced HER2-positive breast cancer. Although it did not significantly improve progression-free survival in the overall population, a trend towards benefit was observed in the PD-L1-positive subgroup [104]. New clinical trials (such as the immunotherapy arm of DESTINY-Breast09) are currently underway [23].

**Combination therapies targeting TAMs:** CSF-1R inhibitors (such as PLX3397 and BLZ945) can reduce M2-type TAM infiltration and restore T-cell function [98,105]. Furthermore, CD47-SIRPα blocking antibodies (e.g., Hu5F9-G4) in combination with trastuzumab are currently being evaluated in early-phase clinical trials [106].

**Reversing ADAR1-mediated immune silencing:** ASO drugs targeting LINC00624 or ADAR1 small-molecule inhibitors are in preclinical development and may, in the future, be combined with anti-HER2 agents for treatment of drug-resistant patients with high ADAR1 expression [102].

**Blocking the HLA-G/KIR2DL4 axis:** Anti-HLA-G antibodies or KIR2DL4 antagonists in combination with trastuzumab have demonstrated the potential to restore ADCC in animal models, warranting further translational research [103].

In summary, immune evasion is a key component of anti-HER2 resistance in HER2-positive breast cancer, and combined immunotherapy strategies targeting TAMs, the ADAR1/IFN pathway, and the HLA-G/KIR2DL4 axis offer new avenues for overcoming resistance. Future research should stratify patients using biomarkers (such as TAM infiltration density, ADAR1 expression levels, and HLA-G positivity rates) to identify those most likely to benefit from combination immunotherapy.

### 3.2 Metabolic Reprogramming and Cell Cycle Abnormalities

In addition to immune mechanisms, adaptive metabolic changes and abnormalities in cell cycle regulation in tumour cells also contribute to the development of drug resistance. This section focuses on SKP2/AKT-mediated glycolysis, impaired ubiquitination and degradation of cyclin D3 in conjunction with the use of CDK4/6 inhibitors, and CXCR4-regulated G2/M phase transition. As direct evidence regarding the mechanism by which lactic acidosis drives adipocyte-to-myofibroblast transdifferentiation (AMT) remains insufficient, please refer to the Supplementary Materials for further details.

#### 3.2.1 SKP2/AKT-Driven Glycolysis (Warburg Effect)

The AKT kinase serves as a key hub linking extracellular signals to cellular metabolism [107,108,109]. Studies have shown that the E3 ubiquitin ligase complex SCF (SKP2-SCF) catalyzes K63-linked ubiquitination of AKT; this modification does not induce AKT degradation but rather promotes its localisation to the cell membrane and its activation via phosphorylation by PDK1/mTORC2. Activated AKT upregulates the expression of the glucose transporter Glut1, enhancing glycolytic flux, which manifests as increased glucose uptake and enhanced lactate production. This metabolic reprogramming provides drug-resistant tumour cells with the ATP and biosynthetic precursors required for rapid proliferation [110].

In HER2-positive breast cancer, high SKP2 expression is closely associated with trastuzumab resistance. Knocking down SKP2 inhibits AKT activation and glycolysis, restoring the sensitivity of tumour cells to trastuzumab. Furthermore, TRAF6, another E3 ubiquitin ligase for AKT, similarly mediates K63 ubiquitination and membrane translocation of AKT upon IGF-1 stimulation [111]. Targeting SKP2 or the glycolytic pathway (e.g., using 2-DG or hexokinase inhibitors) may serve as an adjunctive strategy to overcome resistance.

#### 3.2.2 Impaired Cyclin D3 Degradation and the Use of CDK4/6 Inhibitors

In trastuzumab-sensitive HER2-positive breast cancer cells, trastuzumab promotes the degradation of cyclin D3 via the ubiquitin-proteasome system, inducing cell cycle arrest at the G1/G0 phase. However, in resistant cells, this degradation pathway is impaired, leading to increased cyclin D3 protein stability, which results in the loss of control of cell cycle checkpoints and sustained tumour proliferation [112].

Cyclin D3 is primarily degraded via the Cullin-RING E3 ubiquitin ligase complex (including the SCF complex) [113,114]. Inhibition of the SCF complex or the ubiquitination pathway can further stabilise cyclin D3, thereby exacerbating resistance [112]. CDK4/6 inhibitors (such as palbociclib and abemaciclib) can target the cyclin D3-CDK4/6 complex and have demonstrated potent antiproliferative activity in resistant cells [112,115]. Preclinical studies have confirmed that the combination of abemaciclib and trastuzumab significantly inhibits the growth of resistant tumours; several clinical trials are currently evaluating the efficacy of CDK4/6 inhibitors in combination with anti-HER2 therapy in patients with HER2-positive advanced breast cancer [116].

#### 3.2.3 Regulation of the Cell Cycle and Drug Resistance by the CXCR4 Signalling Axis

The G protein-coupled receptor CXCR4 is highly expressed in various tumours and is involved in chemotaxis, angiogenesis and cell proliferation [117]. In trastuzumab-resistant HER2-positive breast cancer cells, CXCR4 expression is significantly upregulated, and its levels correlate positively with the expression of G2/M transition regulators PLK1, FoxM1 and Wee1. The use of CXCR4 antagonists (such as AMD3100) can downregulate these factors, leading to cell cycle arrest and abnormal mitosis [118].

The expression of CXCR4 is regulated at multiple levels: firstly, the hypoxia-inducible factor HIF-1α transcriptionally activates the CXCR4 promoter; secondly, reduced expression of the E3 ubiquitin ligase ITCH decreases the ubiquitination and degradation of CXCR4; and thirdly, the RNA-binding protein HuR may further enhance its expression by stabilising CXCR4 mRNA. These findings suggest that combined targeting of CXCR4 and its upstream regulatory factors (such as HIF-1α and ITCH) may offer a new approach to reversing trastuzumab resistance [119].

## 4 Challenges and Future Perspectives

Although significant progress has been made in recent years in researching the mechanisms underlying resistance to anti-HER2 therapy in HER2-positive breast cancer—revealing complex mechanisms spanning multiple levels, from the HER2 receptor itself and downstream signalling networks to bypass activation and tumour microenvironment remodelling—numerous challenges remain in the transition from basic research to clinical translation. This chapter will focus on the key scientific issues currently facing the field, emerging therapeutic strategies (using cold atmospheric plasma as an example), and look ahead to future research directions for precisely overcoming resistance.

### 4.1 Core Challenges

#### 4.1.1 Coexistence of Tumour Heterogeneity and Multidrug Resistance Mechanisms

Drug resistance is not driven by a single molecular event, but rather results from the simultaneous or sequential occurrence of multiple mechanisms within the same patient (between or within different tumour sites) or between different patients. For example, some patients may harbour coexisting PIK3CA mutations and PTEN loss, leading to sustained activation of the PI3K/AKT pathway; simultaneously, the infiltration of tumour-associated macrophages (TAMs) and the activation of the HLA-G/KIR2DL4 axis within the tumour microenvironment may synergistically suppress antibody-dependent cell-mediated cytotoxicity (ADCC). This heterogeneity explains why monotherapy often yields limited efficacy. Future therapeutic strategies must be based on a spatiotemporal map of resistance mechanisms, employing multi-target combination or sequential therapies.

#### 4.1.2 The Gap between Preclinical Models and Clinical Reality

Currently, the vast majority of research into drug resistance mechanisms is derived from immortalised cell lines or xenograft models. These models are unable to fully replicate the complex microenvironment of human tumours, the pharmacokinetic characteristics of drugs, or the dynamic interactions with the immune system. Therefore, patient-derived organoids, humanised mouse models, and biomarker-driven prospective clinical trials should be the focus of the next phase of research.

#### 4.1.3 Inconsistent Levels of Evidence and Delays in Assessing Drugability

As demonstrated by the systematic grading in this review, evidence for a large number of mechanisms (such as TRAF4, CMTM6, and NCAPG) remains confined to *in vitro* studies, with only a few (such as PIK3CA mutations and p95HER2) having been clinically validated. The lack of clear stratification regarding the level of evidence and drugability can easily misguide the allocation of subsequent R&D resources. We therefore recommend that future reviews and studies clearly label the validation stage of each mechanism (as ‘clinically validated’, ‘substantial *in vivo* evidence’ or ‘*in vitro* studies only’) and prioritise the clinical translation of those with well-defined drug targeting strategies.

#### 4.1.4 The Potential of Cold Atmospheric Plasma in Drug-Resistant HER2-Positive Breast Cancer

Cold Atmospheric Plasma (CAP) is a physical technology that has emerged in recent years in the field of cancer treatment. By ionising carrier gases (such as helium, nitrogen or air), it generates a plasma jet rich in reactive oxygen species (ROS), reactive nitrogen species (RNS), charged particles, electric fields and ultraviolet radiation, capable of selectively inducing apoptosis, ferroptosis and immunogenic cell death in various tumour cells. Notably, CAP has demonstrated definitive antitumour efficacy in triple-negative breast cancer, a mechanism partly dependent on the regulation of EGFR-mediated signalling pathways.

Given that EGFR bypass activation is one of the core mechanisms underlying resistance to anti-HER2 therapy (particularly trastuzumab and TKIs) in HER2-positive breast cancer, we propose the following hypothetical yet highly translational hypothesis: CAP may reverse resistance in HER2-positive breast cancer via the following pathways [120].

**Inhibition of bypass activation:** ROS/RNS generated by CAP can oxidatively modify EGFR/HER2 receptors or their downstream signalling molecules (such as AKT and ERK), thereby blocking the sustained signal transduction resulting from bypass activation.

**Induction of ferroptosis:** As discussed in this review, resistance to T-DXd is associated with the inhibition of ferroptosis mediated by crVDAC3/HSPB1. CAP is a known potent inducer of ferroptosis, and its combination with T-DXd may synergistically overcome this type of resistance.

**Remodelling the tumour immune microenvironment:** CAP can induce immunogenic cell death (ICD), promote dendritic cell maturation and T-cell infiltration, transforming ‘cold tumours’ into ‘hot tumours’, thereby enhancing ADCC effects and the efficacy of immune checkpoint inhibitors.

Currently, there is a lack of direct studies on CAP in HER2-positive breast cancer resistance models. We strongly recommend conducting preclinical studies to evaluate the efficacy of CAP, either as monotherapy or in combination with T-DXd, trastuzumab, and immune checkpoint inhibitors, in resistant cell lines and patient-derived xenograft (PDX) models. The exploration of this emerging physical therapy may provide the clinical setting with a non-pharmacological, low-toxicity, and reproducible complementary approach to overcoming drug resistance.

### 4.2 Future Perspectives

#### 4.2.1 Real-Time Monitoring of Drug-Resistant Clones Using Liquid Biopsy

Circulating tumour DNA (ctDNA) testing is already used clinically to identify PIK3CA mutations, HER2 copy number variations and truncated receptor mutations (such as p95HER2). In the future, ctDNA should be further integrated with imaging and serum protein biomarkers (such as IL-8 and TGF-β) to establish a multi-parameter dynamic resistance prediction model, thereby achieving a closed-loop management system of ‘treatment-monitoring-adjustment’.

#### 4.2.2 Precision Combination Therapy Strategies Based on Resistance Mechanisms

Rather than blindly pursuing “broad-spectrum” combinations, we should map individual patients’ “resistance profiles” based on multi-region sequencing or single-cell sequencing of tumour tissue. For example, for resistance primarily driven by PIK3CA mutations or PTEN loss, PI3K/AKT inhibitors combined with anti-HER2 therapy should be prioritised; for p95HER2 overexpression or HER3/NRG1 axis activation, irreversible pan-HER TKIs (neratinib, pyrotinib) combined with chemotherapy; and for an immunosuppressive microenvironment characterised by M2-type TAMs or high ADAR1 expression, combination therapy with CSF-1R inhibitors, anti-PD-1/PD-L1 agents or LINC00624 antisense oligonucleotides.

#### 4.2.3 Research and Development of Novel Drug Modalities

In addition to traditional small molecules and antibodies, protein degradation-targeting chimeric proteins (PROTACs) have demonstrated potential in preclinical studies to overcome PI3K/p110α resistance mutations; diversifying the payloads of HER2-targeted ADCs (e.g., DXD, DM1, SN-38) can partially circumvent resistance to single payloads. In the future, the combination of AI-assisted drug design and CRISPR screening is expected to accelerate the discovery of novel interventions targeting ‘undruggable’ targets (such as DUSP6 and NCAPG).

#### 4.2.4 Strengthening Biomarker-Driven Clinical Trial Design

For all future clinical trials aimed at overcoming HER2 resistance, it is recommended that molecular subtyping of resistance mechanisms (e.g., PIK3CA mutation-positive, p95HER2-overexpressing, TAM-high-infiltrating subtypes) be used as a stratification factor for randomisation, in order to evaluate the efficacy gains of personalised combination regimens. Concurrently, patient-reported outcomes (PROs) and pharmacoeconomic assessments should be incorporated to ensure the clinical value and accessibility of new strategies (Table 2).

**Table 2:** Summary of clinical biomarkers and representative clinical trials.

Biomarker	Testing Methods	Predicted Type of Resistance (Drug)	Corresponding Targeting/Mitigation Strategies	Level of Evidence	Representative Clinical Trials
PIK3CA mutation	Tissue NGS/ctDNA	Trastuzumab, Lapatinib, T-DM1	PI3Kα inhibitor	Clinical Validation	NCT05306041 (Phase II)
PTEN deficiency	IHC/FISH	Trastuzumab, T-DM1	AKT inhibitor	Clinical Validation	NCT04305496 (Phase III)
p95HER2 (truncated receptor)	IHC (specific antibody)	Trastuzumab	Irreversible TKI	Clinical Validation	NCT06669975 (Phase I/II)
c-MET amplification/overexpression	IHC/FISH	Trastuzumab	c-MET inhibitor	Clinical Validation	NCT01575522 (Phase II)
HER2 V777L mutation	Tissue/Blood NGS	Trastuzumab	Irreversible TKI + chemotherapy	*In Vitro* Only	No specific tests have yet been conducted
M2-type TAM, highly infiltrated	IHC (CD163, CSF-1R)	Trastuzumab (attenuated ADCC effect)	CSF-1R inhibitor + anti-HER2 therapy	Extensive *In Vivo* Evidence	NCT05491226 (Phase II)
ADAR1 overexpression	IHC/RNA-seq	Trastuzumab, TKIs (solid tumours)	Regorafenib/LINC00624 ASO	Extensive *In Vivo* Evidence	No specific tests have yet been conducted
High expression of HLA-G	IHC/ELISA	Trastuzumab (NK cell inhibition)	HLA-G/KIR2DL4 blocking antibody	Extensive *In Vivo* Evidence	No specific tests have yet been conducted
Iron-dependent death-related (crVDAC3/HSPB1)	IHC/WB	T-DXd	Parirovir + T-DXd	Extensive *In Vivo* Evidence	No specific tests have yet been conducted
High expression of HER3/NRG1	IHC/RNA-seq	Trastuzumab, T-DM1	Anti-HER3 antibody (pertuzumab) + anti-HER2	Extensive *In Vivo* Evidence	NCT05057013 (Phase I/IIa)

***Note.*** 1. Clinically validated: supported by clinical trials or retrospective patient data analysis; 2. Sufficient *in vivo* evidence: validated using transgenic animal models or human xenograft models; 3. *In vitro* studies only: studies conducted solely at the cell line level.

## 5 Conclusion

The mechanisms of resistance to anti-HER2 therapy in HER2-positive breast cancer are complex and diverse, involving intrinsic abnormalities in the HER2 signalling pathway and external remodelling of the tumour microenvironment. Preclinical studies have identified a large number of potential targets, but progress in clinical validation has been relatively slow. As a single mechanism of resistance rarely provides the complete explanation, rational combination regimens should be based on comprehensive molecular pathological testing of tumour tissue, aiming for synergistic targeting of multiple mechanisms rather than single-point inhibition. Concurrently, attention should be paid to the immunological and metabolic regulation of the tumour microenvironment. In addition to targeting HER2 itself, reversing tumour-associated macrophage polarisation, blocking lactate transport, or activating the ADAR1/interferon pathway may all serve as therapeutic breakthroughs following the development of resistance. The development of dynamic monitoring and adaptive treatment strategies is crucial. The use of liquid biopsy and radiomics to track the evolution of resistant clones in real time allows for timely adjustment of treatment regimens, thereby avoiding ineffective treatment and excessive toxicity. Finally, efforts should be made to promote biomarker-driven clinical trial design. Future clinical trials addressing HER2 resistance should incorporate mechanistic subgroups (such as PIK3CA-mutant, p95HER2-overexpressing, and tumour-associated macrophage-rich subtypes) as stratification factors to evaluate the efficacy of personalised combination regimens. In summary, overcoming resistance to anti-HER2 therapy in HER2-positive breast cancer requires a shift from a ‘single-mechanism, static perspective’ to a ‘multi-mechanism, spatiotemporal dynamics’ precision medicine paradigm. The mechanistic classification, evidence differentiation and tabular summaries provided in this review aim to offer clinicians and translational researchers a clear theoretical framework and practical guidance.

## Data Availability

No datasets were generated or analysed during the current study.

## Supplementary Materials

The supplementary material is available online at https://www.techscience.com/doi/10.32604/or.2026.085387/s1.
