# Supplementary material for: Research Advances in Drug Resistance Mechanisms to Anti-HER2 Therapy in HER2-Positive Breast Cancer
Source: Oncol Res. 2026 Jul 16;34(8):10. doi: 10.32604/or.2026.085387 (PMC13397341; doi:10.32604/or.2026.085387)
Supplement: Supplementary file 1 [file OncolRes-34-85387-s001.zip › TSP_OR_85387-s001.docx]

**Supplementary Methods and Results**

**Lactic acidosis drives the conversion of fat cells into myofibroblasts (AMT)**

Research by Elena Andreucci and colleagues has revealed the mechanism by which lactic acidosis in the tumour microenvironment (TME) promotes cancer progression by driving the adipocyte-to-myofibroblast transition (AMT). The study demonstrated that an acidic environment (pH 6.7, containing 10 mM lactate) significantly suppresses adipocyte differentiation markers (such as FABP4, PPARG and ADIPOQ), whilst inducing the expression of myofibroblastic signature genes (such as ACTA2 and COL1A1), thereby promoting the reprogramming of adipocyte precursor cells (acADSCs) into cancer-associated fibroblast (CAF)-like cells. These CAF-like cells secrete pro-inflammatory factors (IL-6, IL-1β) and pro-fibrotic factors (TGF-β1), and through conditioned medium, activate the pro-tumour phenotype of normal fibroblasts whilst enhancing the proliferation, migration, invasion and chemoresistance of breast cancer cells. This mechanism reveals how lactic acidosis reshapes the fate of adipocytes, creating a stromal microenvironment that supports malignant tumour progression [1].

References

1. Andreucci E, Fioretto BS, Rosa I, Matucci-Cerinic M, Biagioni A, Romano E, et al. Extracellular lactic acidosis of the tumor microenvironment drives adipocyte-to-myofibroblast transition fueling the generation of cancer-associated fibroblasts. Cells. 2023;12(6):939. doi:10.3390/cells12060939.
